# First record of the lace bug genus *Eritingis* (Drake and Ruhoff) (Hemiptera: Heteroptera: Tingidae) from Japan and Thailand

**DOI:** 10.3897/BDJ.9.e63188

**Published:** 2021-04-27

**Authors:** Jun Souma

**Affiliations:** 1 Entomological Laboratory, Graduate School of Bioresource and Bioenvironmental Sciences, Kyushu University, Fukuoka, Japan Entomological Laboratory, Graduate School of Bioresource and Bioenvironmental Sciences, Kyushu University Fukuoka Japan; 2 Research Fellowship for Young Scientists (DC1), Japan Society for the Promotion of Science, Tokyo, Japan Research Fellowship for Young Scientists (DC1), Japan Society for the Promotion of Science Tokyo Japan

**Keywords:** Heteroptera, Tingidae, *Eritingis recentis*, lace bug, new record, Japan, Thailand, eastern Asia, Oriental Region

## Abstract

**Background:**

The lace bug genus *Eritingis* Drake and Ruhoff, 1962 is widely distributed in the Australian and Oriental Regions, whereas only a single species, *E. recentis* (Drake and Poor, 1937), has been recorded from the Oriental Region. To date, *E. recentis* is known to occur in Indonesia, Malaysia, Papua New Guinea, Singapore and Vietnam, but has not been recorded from Japan and Thailand.

**New information:**

*Eritingis* and *E. recentis* are recorded from Japan and Thailand for the first time.

## Introduction

The lace bug genus *Eritingis* Drake and Ruhoff, 1962 (Hemiptera: Heteroptera: Tingidae) comprises 11 species from the Australian and Oriental Regions: *E. agyiates* Drake and Ruhoff, 1962, *E. amoena* Drake and Ruhoff, 1962, *E. aporema* Drake and Ruhoff, 1962, *E. exalla* Drake, 1961, *E. hylaea* Drake and Ruhoff, 1962, *E. koebeli* (Drake, 1944), *E. nostratis* (Drake, 1944), *E. pacifica* (Kirkaldy, 1908), *E. recentis* (Drake and Poor, 1937), *E. trivirgata* (Horváth, 1925) and *E. violina* Drake and Ruhoff, 1962 (Drake and Ruhoff 1965a). However, in the Oriental Region, only a single species, *E. recentis*, has been recorded from Malaysia, Singapore and Vietnam to date (Drake and Ruhoff 1965a, Drake and Ruhoff 1965b, Tomokuni 2008).

Recently, I observed a collection of Tingidae from eastern Asia and found an undetermined species of *Eritingis* in Japan and Thailand. After careful morphological examination, I concluded that this undetermined species represents *E. recentis*. In this study, I recorded *E. recentis* for the first time from Japan and Thailand.

## Materials and methods

Dried specimens were used. Morphological characteristics of each specimen were observed under a stereomicroscope (SZ60, Olympus, Tokyo, Japan). Specimens were photographed using a digital microscope (Dino-Lite Premier M, Opto Science, Tokyo, Japan). Distribution records of species were mapped using SimpleMappr (Shorthouse 2010). Geographical coordinates were obtained from Google Map. The terminology used in this study generally follows that of Drake and Ruhoff (1965a) and Schuh and Weirauch (2020). All specimens used for this study were deposited at the Kyushu University Museum, Fukuoka, Japan (KUM).

## Taxon treatments

### Eritingis recentis

(Drake and Poor, 1937)

17A5860E-A6FA-53E8-A37B-081818CA57A4

Perissonemia (Ulonemia) recentis : Drake and Poor 1937a: 5, new species and description.
Perissonemia recentis : Drake and Poor 1937b: 400, distribution.
Ulonemia recens : Drake 1947: 229, new combination and distribution. Tomokuni 2008: 54, unjustified emendation.
Eritingis recens : Drake and Ruhoff 1962: 497, new combination and illustration; Drake and Ruhoff 1965a: 209, catalogue; Drake and Ruhoff 1965b: 255, distribution and illustration.
Eritingis recentis : Tomokuni 2008: 54, distribution.
Perissonemia (Ulonemia) recentis : Drake and Poor 1937a: 5, new species and description.
Perissonemia recentis : Drake and Poor 1937b: 400, distribution.
Ulonemia recens : Drake 1947: 229, new combination and distribution. Tomokuni 2008: 54, unjustified emendation.
Eritingis recens : Drake and Ruhoff 1962: 497, new combination and illustration; Drake and Ruhoff 1965a: 209, catalogue; Drake and Ruhoff 1965b: 255, distribution and illustration.
Eritingis recentis : Tomokuni 2008: 54, distribution.

#### Materials

- **Type status:** Other material. **Occurrence:** recordedBy: Shoichi Miyamoto; individualCount: 2; sex: female; lifeStage: adult; **Taxon:** scientificName: *Eritingis recentis* (Drake and Ruhoff, 1937); namePublishedIn: 1937; kingdom: Animalia; phylum: Arthropoda; class: Insecta; order: Hemiptera; family: Tingidae; genus: Eritingis; specificEpithet: recentis; scientificNameAuthorship: Drake and Poor; **Location:** islandGroup: Ryukyu Islands; island: Okinawa Honto Island; country: Japan; stateProvince: Okinawa; county: Kunigami-son; municipality: Yona; decimalLatitude: 26.75794048; decimalLongitude: 128.22516288; geodeticDatum: WGS84; **Identification:** identifiedBy: Jun Souma; dateIdentified: 2021; **Event:** samplingProtocol: none specified; eventDate: 22-05-1965; **Record Level:** institutionCode: KUM; basisOfRecord: PreservedSpecimen
- **Type status:** Other material. **Occurrence:** recordedBy: Yorio Miyatake; individualCount: 1; sex: female; lifeStage: adult; **Taxon:** scientificName: *Eritingis recentis* (Drake and Ruhoff, 1937); namePublishedIn: 1937; kingdom: Animalia; phylum: Arthropoda; class: Insecta; order: Hemiptera; family: Tingidae; genus: Eritingis; specificEpithet: recentis; scientificNameAuthorship: Drake and Poor; **Location:** continent: Eurasia; country: Thailand; stateProvince: Songkhla; county: Khao Noi; decimalLatitude: 7.22280430; decimalLongitude: 100.56143999; geodeticDatum: WGS84; **Identification:** identifiedBy: Jun Souma; dateIdentified: 2021; **Event:** samplingProtocol: none specified; eventDate: 22-06-1965; **Record Level:** institutionCode: KUM; basisOfRecord: PreservedSpecimen
- **Type status:** Other material. **Occurrence:** recordedBy: Yorio Miyatake; individualCount: 1; sex: female; lifeStage: adult; **Taxon:** scientificName: *Eritingis recentis* (Drake and Ruhoff, 1937); namePublishedIn: 1937; kingdom: Animalia; phylum: Arthropoda; class: Insecta; order: Hemiptera; family: Tingidae; genus: Eritingis; specificEpithet: recentis; scientificNameAuthorship: Drake and Poor; **Location:** continent: Eurasia; country: Thailand; stateProvince: Songkhla; county: Songkhla; decimalLatitude: 7.19543333; decimalLongitude: 100.59582610; geodeticDatum: WGS84; **Identification:** identifiedBy: Jun Souma; dateIdentified: 2021; **Event:** samplingProtocol: none specified; eventDate: 23-06-1965; **Record Level:** institutionCode: KUM; basisOfRecord: PreservedSpecimen

#### Diagnosis

**Figure 1.**
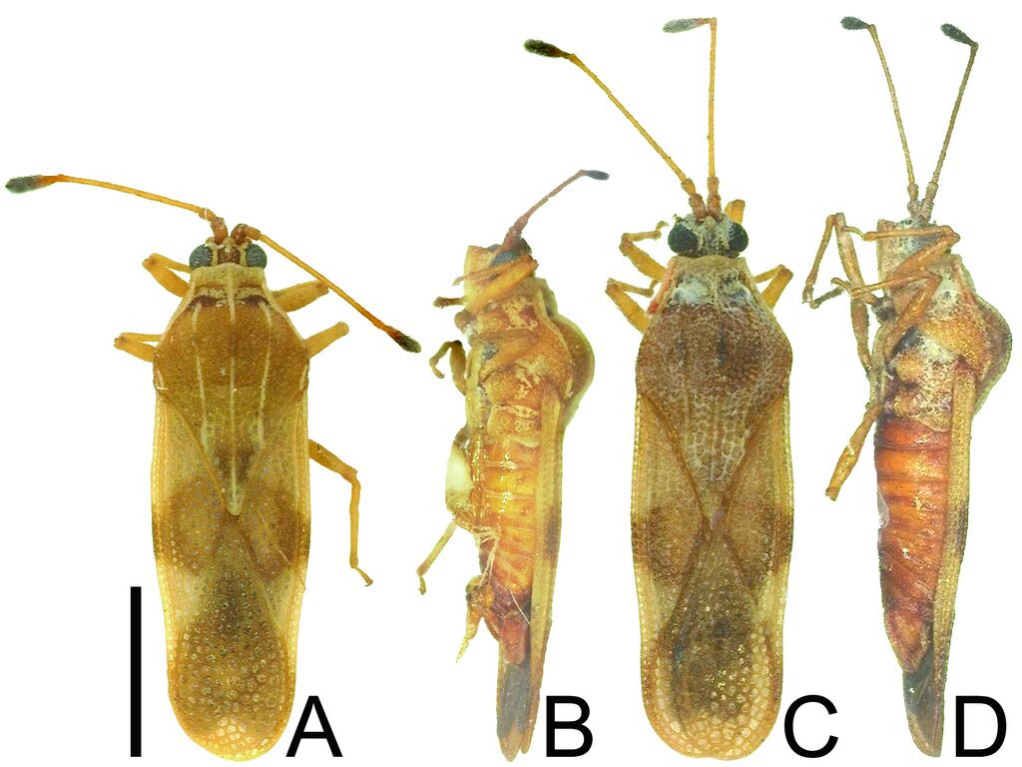
Habitus images of *Eritingis recentis*. **A.** female from Japan, dorsal view; **B.** female from Japan, lateral view; **C.** female from Thailand, dorsal view; **D.** female from Thailand, lateral view. Scale bar 1.0 mm.

**Figure 2.**
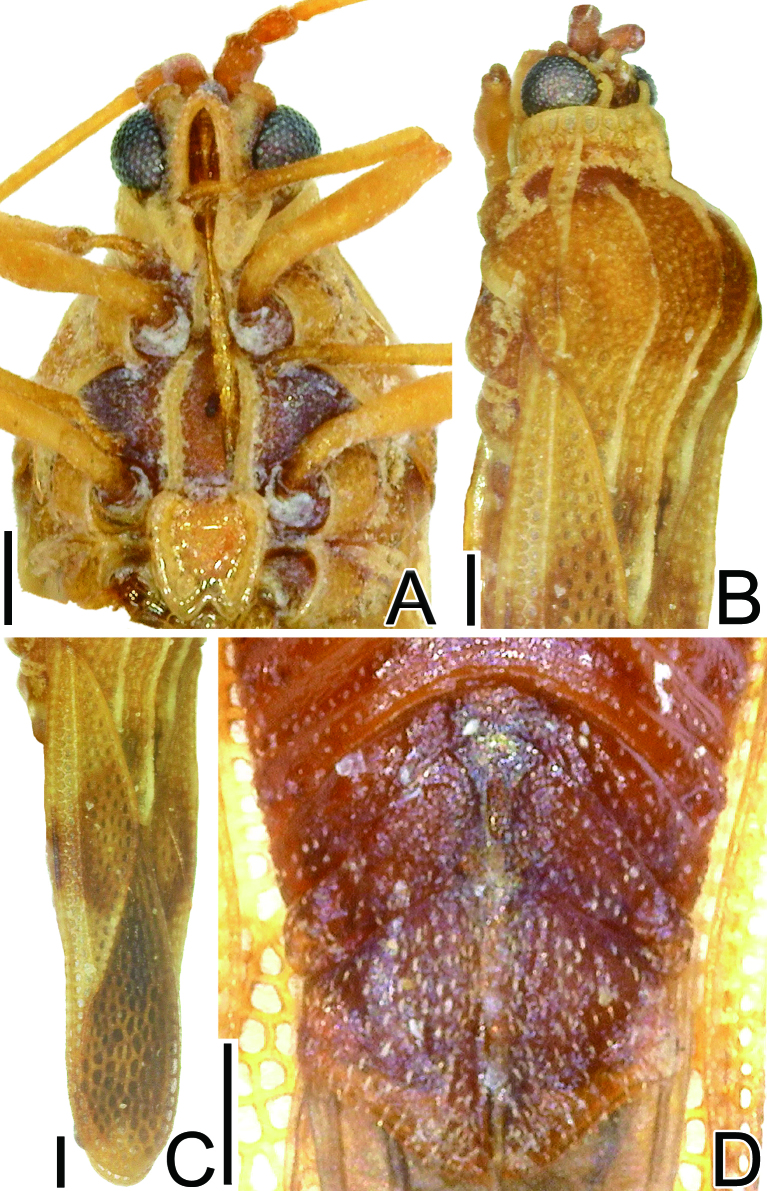
Detailed morphological images of *Eritingis recentis*. **A.** rostrum, ventral view; **B.** pronotum, dorsolateral view; **C.** hemelytron, dorsolateral view; **D.** female terminalia, ventral view. Scale bars 0.2 mm.

*Eritingis recentis* can be distinguished from other species of *Eritingis* based on a combination of the following characteristics: body length 3.0–3.2 mm, 3.3 times as long as maximum width across hemelytra (Fig. 1a–d); rostrum reaching anterior margin of metasternum (Fig. 2a); anterior margin of hood protruding anteriad in middle part (Fig. 2b); anterior margin of hemelytron nearly straight (Fig. 2c); costal area distinct, with a single row of areolae throughout its length; subcostal area with 1–2 rows of areolae in basal part and 2 rows in remaining parts; discoidal area with 5–6 rows of areolae at widest part; sutural area with 9 rows of areolae at widest part; and female terminalia pentagonal in ventral view (Fig. 2d).

#### Distribution

**Figure 3.**
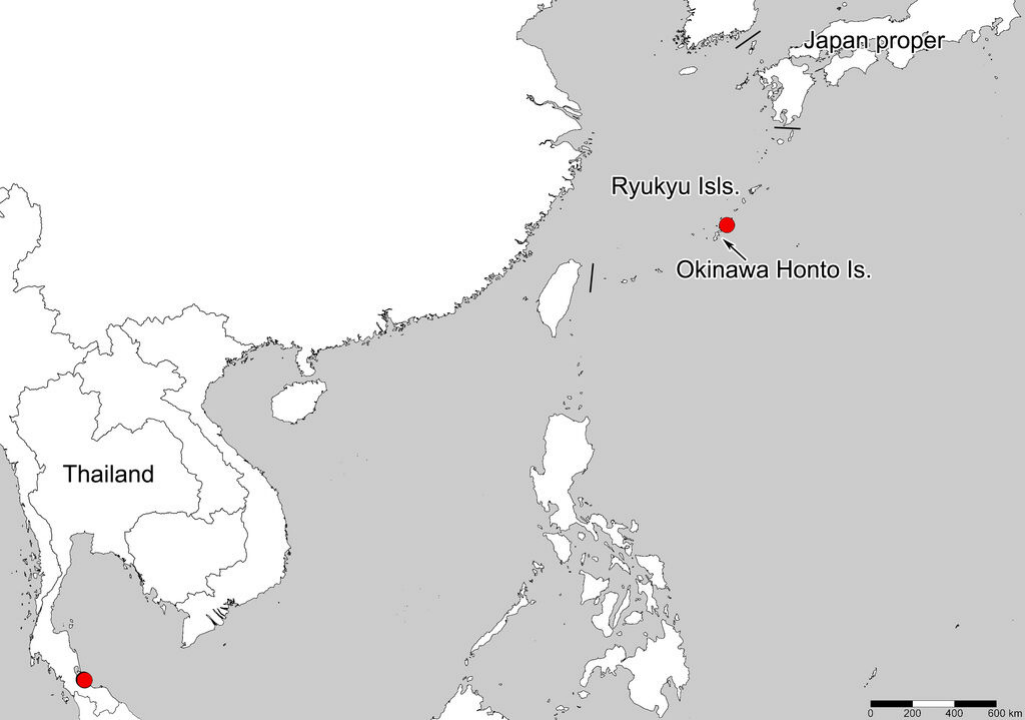
Collection sites of *Eritingis recentis* in Japan and Thailand examined in present study.

Japan (Ryukyu Islands: Okinawa Honto Is.) (Fig. 3), Indonesia, Malaysia, Papua New Guinea, Singapore, Thailand, Vietnam (Drake and Ruhoff 1965a, Drake and Ruhoff 1965b, Tomokuni 2008, present study).

The discovery of *Eritingis recentis* from Japan represents the northernmost distributional record of *Eritingis* species.

#### Biology

Adults have been collected in almost all seasons (Drake and Ruhoff 1965b, Tomokuni 2008, present study). The host plant for *Eritingis recentis* is unknown (Drake and Ruhoff 1965a, Tomokuni 2008).

#### Taxon discussion

The above recorded specimens match well with the photographs of the holotype (United States National Museum of Natural History 2021), illustrations (Drake and Ruhoff 1962, Drake and Ruhoff 1965b) and original description (Drake and Poor 1937a) of *Eritingis recentis* described from Singapore.

*Eritingis recentis* is highly similar to *E. agyiates* in general appearance. The latter is distinguished from the former by the body length 2.7 mm and the rostrum reaching the anterior margin of mesosternum.

## Supplementary Material

XML Treatment for Eritingis recentis
